# Associations of solution building and resilience with school refusal behavior among elementary school students

**DOI:** 10.1371/journal.pone.0356881

**Published:** 2026-08-24

**Authors:** Takefumi Yoshida, Koubun Wakashima, Gen Takagi, Mayumi Sakuraba, Sara Smock Jordan, Kazuma Sakamoto, Ranno Haruyama

**Affiliations:** 1 Hachihonmatsu Elementary School, Sendai, Japan; 2 Graduate School of Education, Tohoku University, Sendai, Japan; 3 Faculty of Humanities and Social Sciences, Shizuoka University, Shizuoka, Japan; 4 Iwaki Junior College, Iwaki, Japan; 5 University of Nevada Las Vegas, Las Vegas, United States of America; 6 Graduate School of Education, Tohoku University, Sendai, Japan; 7 Graduate School of Human Sciences, Osaka University, Osaka, Japan; Sreenidhi Institute of Science and Technology, INDIA

## Abstract

This study examined the effects of solution building and resilience on school refusal behavior. The participants were 587 elementary school students (280 boys, 306 girls, and one student who did not report gender) who completed a self-report questionnaire. The results showed that resilience skills had a direct negative effect on the intensity of school refusal behavior among boys and an indirect negative effect mediated by solution building. However, among girls, resilience skills did not directly affect the intensity of school refusal behavior, but had a negative indirect effect through solution building. These findings suggest that school refusal behavior is less intense among children with higher resilience and strong solution-building skills. This study provides evidence that may inform the development of effective support strategies aimed at reducing the intensity of school refusal behavior.

## Introduction

According to the “Survey on Student Behavioral Problems, School Non-attendance, and Other Issues in Student Guidance, Fiscal Year 2024” [1], the number of students refusing to attend school in elementary and junior high schools has reached 353,970 in Japan. This number has increased over 12 consecutive years, reaching an all-time peak. Therefore, it is important to address school refusals. In addition, it has been revealed that many students who currently attend school want to avoid going [2–5]. Therefore, school refusal tendency among school-attending students should also be considered as an important target for preventive support. Methods to prevent this should be considered by providing support during the attendance stage [6]. However, at present, support has not yet reached the preschool refusal stage and preventive support is crucial [7]. Therefore, to prevent school refusal, ways to prevent children’s maladjustment and foster their abilities to overcome stressful situations are necessary [8].

### Present status of the school refusal tendency

Research is underway on students who express a desire to avoid school as well as the related factors that contribute to this phenomenon. Morita [2] defined the sentiment of reluctance to attend school as “school-avoidance feelings,” irrespective of actual absenteeism, and posited that the frequency of these sentiments contributes to school refusal. Nakamura et al. [4] conducted an analysis of the “Survey on Children’s Living Conditions” that was conducted in Shizuoka Prefecture in fiscal year 2003. The results indicated that the proportion of children exhibiting a tendency toward school refusal, defined as those who reported that they “often felt unwilling to go to school (about once a week),” was approximately 10% among boys in both elementary and junior high school, around 10% among girls in elementary school, and as high as 20% among girls in junior high school. Tsuchiya et al. [9] found that approximately 27% of children often feel that they “want to do something different from that at school without attending school.” Igarashi [5] showed that approximately 27% of elementary and 32% of junior high school students answered, “I sometimes go to school even if I don’t want to.” Ueji and Takakura [3] reported that 21.7% of students expressed sentiments indicative of school avoidance, suggesting that a significant number of students attending school may not desire to do so.

Igarashi and Hagiwara [10] defined “school refusal tendency” as a precursor to school refusal in which students have difficulty enjoying school life. School refusal tendency in elementary school has correlated with the same tendency in junior high school. Igarashi [6] reported a weak to moderate significant positive correlation using a longitudinal study. Therefore, reducing school refusal tendencies at elementary level is expected to prevent an increase in school refusal in junior high school.

Meanwhile, Kearney and Silverman [11] defined “school refusal behavior” as “absenteeism from school and difficulty going to or staying in school.” They viewed this as a continuous concept, ranging from total absence to behaviors exhibited by students who still attend school. Kearney and Silverman [12] examined the maintenance factors of school refusal behavior from the perspective of a functional analytic model. They assumed four functions: 1) avoidance of negative affectivity-provoking objects or situations related to a school setting, 2) escape from aversive social or evaluative situations, 3) attention-getting behavior, and 4) positive tangible reinforcement [9]. Tsuchiya et al. [9] clarified that this model also applies to children currently attending school. To prevent total absence, addressing school refusal behavior exhibited by children attending school is crucial [13]. The present study did not focus on clinically identified school refusal; rather, school refusal behavior was examined as a continuous construct among school-attending children. Thus, this study focuses on “school refusal behavior” [11] as a continuous concept to examine measures for children who attend school but exhibit a tendency toward school refusal. Notably, the state preceding complete non-attendance is termed “school refusal tendency.”

### Factors related to school refusal tendency

Various factors related to school refusal tendency have been examined [4,10,14]. One such factor is psychological stress experienced in school life [15,16]. Shimada [17] reported that students experiencing strong school-related stress have low motivation to attend school, and those exhibiting stress reactions show high subjective school maladjustment. Igarashi [6] pointed out that students with a “play-seeking school refusal tendency” often struggle to seek help or regulate stress. Furthermore, Kobayashi and Igarashi [18] showed that students with high school refusal tendency in the 8th grade generally exhibited higher stress responses throughout grades 4–8. Igarashi and Kobayashi [19] conducted a longitudinal study of fourth to sixth graders and demonstrated that students with lower school refusal tendencies in elementary school also exhibited lower stress responses. To prevent maladaptive states and maintain adaptive states, mitigating psychological damage from stressors are required [8]. Therefore, acquiring stress-coping methods from elementary school may contribute to reducing school refusal tendencies.

The psychological characteristic of maintaining or recovering mental health in stressful situations is called “resilience” [20]. A link between school refusal tendencies and resilience has been demonstrated [21]. Kobayashi et al. [22] reported that, among resilience factors, the ability to consider difficult situations from various angles and remain calm particularly weakens the school refusal tendency. Kobayashi [23] indicates that a relationship exists between school refusal tendencies and resilience. Objective perspectives that consider matter from multiple perspectives can suppress children’s school refusal tendencies. Furthermore, resilience skills developed during the elementary school years are associated with school refusal tendencies during junior high school. Therefore, enhancing children’s resilience may reduce school refusal tendency and contribute to its prevention. Kobayashi et al., [24] state that previous studies share the common perspective of conceptualizing learnable cognitive and behavioral protective factors related to resilience as “resilience skills.” Protective factors are elements that positively modify the impact of risks, even when risks are present. They pointed out that examining the protective factors of resilience from this perspective provides foundational insights into fostering children’s resilience. Therefore, this study defines and addresses resilience skills as “protective factors that promote the process of recovering to a better adaptive state after experiencing difficulties, viewed from cognitive and behavioral aspects” [24].

### Toward reducing school refusal tendency

Support focusing on resilience and its utilization is required to enhance children’s ability to cope with stress and reduce school refusal tendencies. In exploring the relationship between resilience and school refusal tendency, this study focuses on the potential role of “solution building” as a mediator. “solution building” is a key concept in solution-focused brief therapy [25]. Smock et al. [26] defined solution building as consisting of three aspects: clearly identifying the solution [27], increasing the client’s awareness of exceptions [27,28], and developing hope for the future [29].

Regarding school refusal due to stress, Sakuraba et al. [13] suggested that an intervention using the SFBT may reduce the factors that maintain school refusal behavior. This approach involves exploring solutions to problems based on individual goals, strategies, strengths, and resources. Sakuraba et al. [30] reported that junior high school students who participated in SFBT-based workshops tended to prefer staying with family to going to school. Therefore, high solution building is presumed to help reduce school refusal tendencies. Furthermore, solution building has been suggested to be associated with resilience. Grant et al. [31] found that solution-focused thinking—specifically goal orientation, resource activation, and disengagement from problems—showed a positive correlation with resilience and a negative correlation with problem orientation. This finding suggests that increased resilience may enhance solution building. In other words, higher resilience is expected to lead children to adopt more adaptive coping behaviors related to solution building, resulting in reduced school refusal.

However, it remains unclear how high solution building and resilience skills in elementary school children specifically influence school refusal behaviors. Research findings targeting elementary school children are insufficient and it is necessary to clarify how children’s solution-building and resilience skills relate to school refusal tendencies.

### Purpose of this study

This study aimed to examine the associations of solution building and resilience with school refusal behavior among elementary school students. This study was based on the assumption that students who exhibit high levels of both solution-building and resilience skills demonstrate lower school refusal tendencies. The hypothesis predicts that resilience skills directly influence solution building positively and indirectly influence school refusal behavior negatively through the mediating effect of solution building.

## Methods

### Procedure

The survey was conducted from January 13, 2025 to March 31, 2025. Permission to conduct the survey was obtained from the school principal. During distribution, the survey was explained in writing and responses were requested only after obtaining parental consent, ensuring informed consent. Participants were advised that if answering the survey caused distress, such as by recalling painful experiences, or if they were currently troubled by such issues, they should consult their homeroom teacher or school counselor, demonstrating consideration for potential harm. The survey was approved by the Research Ethics Review Committee of the Graduate School of Education, Tohoku University (Approval ID: 24-1-051).

### Participants

A questionnaire survey was administered to 600 elementary school students (grades 4–6). After excluding incomplete responses, 587 students (280 boys, 306 girls, and one unknown; mean age = 10.83, *SD* = 0.86) were analyzed.

### Questionnaire

The questionnaire comprised the sections as set out as follows.

#### Demographic.

Participants were asked about their age, sex, class, and ID number.

#### Solution building.

The Solution Building Inventory for Children (SBI-C) [32] was used. It consists of 14 items, a 5-point scale, and a single-factor structure.

#### Resilience.

The Resilience Skills Scale for Teenage Children [24] was used. It consists of 17 items, a 5-point scale, and a four-factor structure: “Calm relations with self and others” “Positive Self-Awareness,” “Routine Behavior,” and “Self-Care.”

#### School refusal behavior.

The Japanese version of the School Refusal Assessment Scale-Revised for School-Attending Children (SRAS-R-JA) [9] was used. It consists of 20 items on a 5-point scale and has a 4-factor structure: “avoidance of stimuli that provoke negative affectivity,” “escape from aversive social and/or evaluative situations,” “pursuit of attention from significant others,” and “pursuit of tangible reinforcement outside the school setting.” Tsuchiya et al. [9] conducted a Kruskal-Wallis test with the independent variable being school refusal experience and the dependent variable being the total score on the SRAS-R-JA, reporting significant differences. Therefore, this study used the total SRAS-R-JA score to analyze school refusal behaviors.

## Results

### Scale scoring and reliability

The mean values for each scale were calculated, scores were assigned, and alpha coefficients were computed. The results showed sufficient values above .80 for all scales except Routine Behavior (.72) and Self-Care (.69): Solution Building (.89), Calm Interaction with Self and Others (.82), Positive Self-Awareness (.86), Routine Behavior (.72), Self-Care (.69), and SRAS-R-JA (.93). The Self-Care subscale demonstrated a Cronbach’s α coefficient of .69, which was slightly below the conventional criterion of .70. However, the item content was carefully reviewed and showed conceptual coherence. Therefore, this subscale was retained for subsequent analyses. The scale scores were calculated and used directly in the analyses (Table 1).

**Table 1:** Descriptive statistics of scale.

	M	SD	MIN	MAX	Cronbach’s α
SBI-C	52.71	9.10	14	70	.89
CRWSO	31.14	5.66	9	40	.82
PSA	15.75	3.98	4	20	.86
RB	9.72	3.45	3	15	.72
SC	7.25	2.44	2	10	.69
SR	40.56	14.36	20	96	.93

SBI-C: Solution Building Inventory for Children, CRWSO: Calm relations with self and others, PSA: Positive Self-Awareness, RB: Routine Behavior, SC: Self-Care, SR: School Refusal.

### Gender differences

To examine the sex differences between variables, an independent t-test was conducted. The results showed that female participants scored higher than male participants on the SBI-C (*t* (584) = −2.76, *p* < .01). Regarding resilience skills, female participants scored higher than male participants on “Self-Care” (*t* (569.52) = −2.093, *p* < .05) (Table 2).

**Table 2:** Descriptive statistics and the results of independent-samples t-tests by gender.

	Male		Female		*t*	*p*
	M	SD	M	SD		
SBI-C	51.65	9.77	53.72	8.34	-2.76**	0.006
CRWSO	30.79	5.98	31.46	5.35	-1.44	0.152
PSA	15.64	4.18	15.85	3.81	-.62	0.537
RB	9.45	3.62	9.97	3.28	-1.83	0.068
SC	7.03	2.52	7.45	2.35	-2.09*	0.037
SR	40.94	14.73	40.10	13.90	.71	0.477

*p<.05, **p<.01,***p<.001

### Correlation analysis

The results of the correlation analysis are presented in Table 3. Solution Building showed a significant negative correlation with school refusal behavior. Resilience skills showed significant negative correlations with school refusal behavior for all four factors Furthermore, Solution Building showed significant positive correlations with all four resilience skills. Significant positive correlations were found among the four resilience skills.

**Table 3:** Correlations .

	1	2	3	4	5
1. SBI	—				
2.CRWSO	.74***	—			
3. PSA	.65***	0.63***	—		
4. RB	.39***	0.44***	0.35***	—	
5. SC	.39***	0.41***	0.34***	0.34***	—
6. SR	-0.34***	-0.33***	-0.32***	-0.16***	-0.09*

*p<.05, **p<.01,***p<.001

### Mediation analysis

We conducted indirect effect tests using the bootstrap method (bootstrap count: 5,000) with JASP (version 0.18.3) to examine indirect effects. An indirect effect is considered significant when the 95% confidence interval obtained from the bootstrap method does not include zero [33]. Because gender differences were observed in Solution Building and Self-Care, we tested the indirect effects of resilience skills on school refusal behavior mediated by Solution Building separately for boys and girls.

First, among boys, “Positive Self-Awareness,” “Routine Behavior,” and “Self-Care” were indirectly and negatively associated with school refusal behavior through the mediation of Solution Building. However, the indirect effect of “Calm relations with self and others” mediated by Solution Building on school refusal behavior was not significant. Furthermore, only “Calm relations with self and others” and “Positive Self-Awareness” exhibited direct effects on school refusal behavior (Table 4).

**Table 4:** Examination of Direct and Indirect Effects (boys).

Pathway					Effect Size	SE	95% CI	
							Lower	Upper
Direct Effects								
CRWSO	→	SR			-0.563	0.201	-1.03	-0.08
PSA	→	SR			-0.903	0.252	-1.406	-0.331
RB	→	SR			-0.277	0.244	-0.799	0.26
SC	→	SR			0.684	0.349	-0.04	1.365
Indirect Effects (via SBI)								
CRWSO	→	SBI	→	SR	-0.302	0.149	-0.725	0.02
PSA	→	SBI	→	SR	-0.386	0.164	-0.785	-0.069
RB	→	SBI	→	SR	-0.445	0.111	-0.73	-0.226
SC	→	SBI	→	SR	-0.771	0.173	-1.228	-0.428

Next, for girls, all four resilience factors were indirectly negatively related to school refusal behavior through Solution Building. Additionally, none of the factors had a direct effect on school refusal behavior (Table 5).

**Table 5:** Examination of Direct and Indirect Effects (girls).

Pathway					Effect Size	SE	95% CI	
							Lower	Upper
Direct Effects								
CRWSO	→	SR			-0.259	0.214	-0.848	0.289
PSA	→	SR			-0.274	0.268	-0.842	0.265
RB	→	SR			-0.009	0.253	-0.497	0.515
SC	→	SR			-0.087	0.326	-0.717	0.567
Indirect Effects (via SBI)								
CRWSO	→	SBI	→	SR	-0.527	0.163	-0.961	-0.101
PSA	→	SBI	→	SR	-0.724	0.187	-1.165	-0.343
RB	→	SBI	→	SR	-0.608	0.130	-0.936	-0.356
SC	→	SBI	→	SR	-0.844	0.182	-1.273	-0.505

## Discussion

This study examined whether resilience influences school refusal behavior by mediating Solution Building. The hypothesis predicted two things: first, that resilience would directly and positively affect solution building, and second, that resilience would indirectly and negatively affect school refusal behavior through the mediating effect of solution building.

### Impact of solution-building and resilience skills on school refusal behavior

In this study, for boys, “Calm relations with self and others” and “Positive Self-Awareness” directly impacted school refusal behavior. Specifically, controlling one’s emotions to think calmly when faced with difficulties or focusing on one’s positive attributes reduced school refusal behavior. Additionally, “Positive Self-Awareness,” “Routine Behavior, and “Self-Care” had an indirect negative effect on school refusal behavior mediated by Solution Building. For girls, all resilience skills had an indirect negative effect on school refusal behavior, which was mediated by Solution Building. In other words, as resilience skills increased, Solution Building improved, and consequently school refusal behavior decreased. Therefore, the hypothesis is largely supported.

Grant et al. [31] and Takagi et al. [34] demonstrated a positive association between solution building and resilience. In this study, solution building was positively correlated with resilience skills. Furthermore, higher resilience among elementary school students reduces school refusal tendency [22,23]. Additionally, among junior high school students, when solution building is high, the higher the resilience, the lower the functional significance of school refusal behavior [13].

However, it remains unclear how solution building and resilience influence school refusal behavior among elementary school students. The results of this study revealed that among elementary school students with high resilience skills, higher solution building was associated with fewer school refusal behaviors. Specifically, for boys, higher solution building was associated with fewer school refusal behaviors when they possessed higher resilience skills related to focusing on their positive aspects, creating and adhering to their own daily routines, and acquiring and practicing conscious relaxation techniques. For girls, in addition to the above, higher solution building was associated with fewer school refusal behaviors when they had higher resilience skills related to calmly assessing situations, understanding others’ perspectives, and overcoming difficult situations by changing their mindset. In summary, this study found that among elementary school students with high resilience skills, higher solution building was associated with fewer school refusal behaviors.

Furthermore, in this study, only two resilience skills, “Calm relations with self and others” and “Positive Self-Awareness,” had a direct effect on school refusal behavior among boys. In contrast, for girls, resilience skills exerted a negative influence on school refusal behavior through the complete mediation of solution building. This difference may stem from differences in stress-coping strategies between boys and girls. Previous research has indicated that girls tend to actively seek social support and engage in problem-solving behaviors more frequently than boys do [35]. Furthermore, Ohtake et al. [36] reported that girls more frequently use coping strategies such as “problem-solving” (attempting to resolve problems through their own efforts) and “seeking support” (requesting help or cooperation from others to solve problems) as coping strategies.

Based on these findings, it is believed that higher resilience skills do not directly influence school refusal behavior in girls. Rather, school refusal behavior may be reduced through actual coping actions and the selection and implementation of appropriate strategies. Solution building is a central concept of Solution-Focused Brief Therapy and involves defining a preferred future, identifying exceptions to the problem, and expanding hope for the future. These processes require individuals to reflect on their experiences and become aware of available resources. Therefore, for girls, psychological resources derived from resilience may influence school refusal behavior through solution-building processes, such as constructing a preferred future and recognizing available resources.

In contrast, the direct effects observed among boys may be related to gender-specific coping tendencies. Hampel and Petermann [37] reported that boys are more likely than girls to use “positive self-instructions,” a cognitive self-regulatory strategy involving encouraging or reassuring oneself when facing difficult situations. Furthermore, although positive self-instructions are theoretically classified as a problem-focused coping strategy, they have also been suggested to include emotion-regulation functions [38].

The resilience skill “Positive Self-Awareness,” which showed a direct effect in the present study, reflects a tendency to focus on one’s strengths and positive attributes, whereas “Calm relations with self and others” includes characteristics related to emotional regulation and adaptive interpersonal functioning. Therefore, these resilience skills may be closely associated with the cognitive and emotional self-regulatory processes represented by positive self-instructions. Consequently, among boys, some resilience skills may contribute to reducing school refusal behavior not only through solution building but also through cognitive and emotional self-regulation processes.

Therefore, enhancing both resilience skills and solution building may be an effective approach for reducing children’s school refusal behavior. The findings of this study are expected to contribute to future discussions on effective support strategies for reducing school refusal behavior.

### Limitations of the present study and future research

This study has several limitations that should be considered when interpreting the findings. First, this study employed a cross-sectional observational design. Therefore, causal relationships among resilience, solution building, and school refusal behavior cannot be established. Furthermore, it remains unclear whether enhancing resilience and solution-building skills would reduce school refusal behavior over time. Future longitudinal and intervention studies are needed to examine these causal relationships and intervention effects.

Second, all variables were assessed using self-report questionnaires. Therefore, the findings may have been influenced by response biases, such as social desirability or recall bias. Future studies should incorporate multiple informants, including parents and teachers, as well as behavioral indicators of school attendance.

Third, the participants were elementary school students from Japan. Therefore, caution is warranted when generalizing the findings to students from different cultural backgrounds or educational systems. Future cross-cultural studies are needed to examine the universality of the present findings.

Fourth, potentially important variables related to school refusal behavior, such as socioeconomic status, family functioning, academic achievement, bullying experiences, anxiety, and depressive symptoms, were not assessed in the present study. Future studies should examine these factors simultaneously to better understand the mechanisms underlying school refusal behavior.

Fifth, although mediation analyses were conducted separately for boys and girls, formal multi-group structural equation modeling analyses were not performed. Therefore, gender differences in the mediation pathways were not statistically compared. Future studies should employ formal multi-group SEM analyses to investigate gender differences more rigorously.

Sixth, this study was conducted within the Japanese educational and cultural context using instruments validated for Japanese children. Therefore, caution is warranted when generalizing the findings to other countries and educational systems. Future studies should examine the generalizability of the present findings across different cultural and educational contexts.

Seventh, the present study assessed school refusal behavior as a continuous construct among children who were attending school and did not include children who were currently experiencing school refusal. Therefore, the generalizability of the present findings to clinically or practically identified school refusal populations remains uncertain. Future studies should examine whether the observed relationships can be replicated among children who are currently experiencing school refusal.

This study demonstrates that resilience skills indirectly influence school refusal behavior through solution building. However, to establish that resilience skills and solution building reduce school refusal tendencies, it is necessary to verify the effectiveness of interventions aimed at enhancing solution building. Future research should accumulate empirical evidence through intervention studies showing that such interventions effectively enhance resilience skills and solution building, thereby reducing school refusal tendencies in both the short and long term. Therefore, developing programs are necessary for elementary school students to enhance their resilience skills and solution building to reduce school refusal behaviors.

Considering interventions based on solution-focused brief therapy to enhance resilience skills and solution building in elementary school students and reduce school refusal tendencies can be considered a step toward preventing school refusal issues.

## Conclusion

This study examined the effects of solution-building and resilience skills on school refusal behavior among elementary school students. The results showed that certain resilience skills directly reduced school refusal behavior in boys. These skills included “Positive Self-Awareness” and “Calm relations with self and others.” Other skills, such as “Positive Self-Awareness,” “Routine Behavior,” and “Self-Care,” had indirect negative effects mediated by solution building. In girls, all resilience skills indirectly reduced school refusal behavior through solution building, with no direct effects observed. These findings suggest that higher levels of resilience skills, when accompanied by high solution-building skills, are associated with lower levels of school refusal behavior. Furthermore, sex differences in patterns of influence suggest that coping strategies may moderate these effects. Overall, enhancing resilience skills and solution building appear to be promising approaches for preventing school refusal behavior and developing early intervention programs in elementary school settings.

## Supporting information

S1 Data Dataset used for statistical analyses. (XLSX)
